# CT Perfusion imaging as prognostic factor for outcome of lacunar stroke

**DOI:** 10.1007/s00234-024-03480-2

**Published:** 2024-10-10

**Authors:** Stefan Mausbach, Lamya Ahmad Abdallah, Eliel Ben-David, Michael Teitcher, Natan M. Bornstein, Roni Eichel

**Affiliations:** 1 Department of Neurology, Shaare Zedek Medical Center and Faculty of Medicine, Hebrew University of Jerusalem, Shmuel Bait 12, Jerusalem, 9103102 Israel; 2 2Department of Neurocritical Care Medicine, Shaare Zedek Medical Center and Faculty of Medicine, Hebrew University of Jerusalem, Jerusalem, Israel; 3 Department of Radiology, Shaare Zedek Medical Center and Faculty of Medicine, Hebrew University of Jerusalem, Jerusalem, Israel

**Keywords:** Cerebral blood flow, Cerebrovascular reserve, Lacunar stroke

## Abstract

**Background:**

Early neurological deterioration (END) affects 20–30% of patients with lacunar stroke within 48 h despite optimal treatment. Previously established markers included infection and infarct location on imaging. We studied the utility of measuring global cerebral blood flow (gCBF) measured by CT-Perfusion (CTP) as an early predictor of END in patients with lacunar strokes.

**Methods:**

162 patients with lacunar stroke were measured for gCBF including both cerebral hemispheres and cerebellum. We stratified patients by normal gCBF (> 40 ml/100 mg/min) vs. low gCBF (< 40 ml/100 mg/min). Stroke location, vascular risk factors, age and gender were assessed. The primary outcome was the change in the NIHSS score after 48 h from index stroke.

**Results:**

Mean gCBF of the overall cohort was 37.72 ml/100 mg/min. Both groups had a baseline NIHSS score of 4.2 with similar standard deviations. The NIHSS score decreased by 1.3 points in normal gCBF group and increased by 1.1 points in the low gCBF group. All stroke sites deteriorated in the low gCBF group, particularly the capsula interna, corona radiata, and lateral pontine area. END occurred in 37.8% in low gCBF compared to 3.1% in the normal gCBF patients. In contrast, clinical improvement after 48 h occurred in 64.2% of patients with normal gCBF but only 6.1% with low gCBF.

**Conclusion:**

Our study supports measurement of gCBF by CTP as a potential imaging biomarker for END. Additionally, it adds evidence to the body of supporting the vulnerability of capsula interna and pontine infarctions to END.

## Introduction

Lacunar stroke is defined as small vessel ischemia and was first described in 1883 by Dechambre. The word originates from Latin with the meaning “hole.” The STRIVE consortium [1] defines a lacunar stroke radiologically as a round or ovoid subcortical cavity measuring 3 to 15 mm, not exceeding 20 mm. Lacunar strokes are estimated to account for 20–30% of all strokes [2]. The recurrence rate is around 20%, and up to 50% of all healthy elderly individuals show silent lacunar infarctions on imaging [3]. The aetiology is not fully understood but includes multiple explanations. Hypertensive microangiopathy [4] is causing lipohyalinosis with subsequent small vessel thickening and smooth muscle involvement. This causes a significant lumen reduction to less than 200 μm in diameter. Additional factors are diabetes mellitus and hyperlipidemia. The narrowing causes hypoperfusion and ultimalty a lacunar infarction. Microatheroma [5], typically located in the proximal region of a deep penatrating artery causes stenosis or occlusion. They are indistinguishable from large vessel atheroma. Atheroma are filled with lipids, fibroblasts and macrophages. Embolism [6], either direct emboli by a cardiac thrombus or large artery to artery source are less common. Lesser known factors are cerebral autosomal dominant arteriopathy with subcortical infarcts (CADASIL) and lipid storage diseases. A new, yet unconfirmed explanation is endothelial dysfunction causing capillary edema [1]. Approximately 20–30% of lacunar strokes exhibit early neurological deterioration (END), often defined as worsening of at least two points on the National Institute of Health Stroke Scale (NIHSS) [7, 8].

There are limited predicting factors and biomarkers for outcome of lacunar infarct. Studies [9, 10] indicate that C-reactive protein (CRP) or early signs of infection can serve as predictors. Vynckier et al. [7] demonstrated that prognostic markers specifically include the capsular warning sign, infarct site, and hypoperfusion lesion on CT Perfusion (CTP) imaging.

One essential marker for autoregulation is Cerebrovascular Resistance (CVR), but its exact measurement remains problematic. Estimates can be calculated as CVR = MAP/CBF, where Mean Arterial Pressure (MAP) and CBF are obtainable through neuroimaging techniques. Cerebral autoregulation can be divided into two systems: static and dynamic autoregulation. In 1959, Lassen [11] demonstrated that cerebral autoregulation maintains a constant CBF for changes in Mean Arterial Pressure (MAP) between 60 and 170 mmHg. Aaslid et al. [12] proposed a model of dynamic autoregulation showing a drop in CBF after an acute reduction of MAP, with autoregulation starting after a delay. Generally, healthy individuals exhibit an average CBF of 40 to 50 ml/100 mg/min [13]. These findings emphasize the significance of understanding autoregulation mechanisms for better clinical outcomes.

CTA should theoretically offer a good estimate of collateral flow, but studies like MR CLEAN LATE [14] and the Guidelines from Stroke and Vascular Neurology for measuring collateral flow show less reliability for CTA specifically for hypoplastic segments with a sensitivity of 53% [15]. Other studies like DAWN [16] or Defuse-3 [17] show a benefit of CTP with good estimation of collateral flow. CTP is readily available on admission of stroke patients in most stroke centers and allows measurement of CBF in restricted areas as well as globally.

Considering the relationship between CRV and CBF and their potential impact on lacunar strokes, our study focused on utilizing CTP as a promising prognostic tool for evaluating the outcome of lacunar strokes. By gaining insights into these mechanisms, we aim to enhance our understanding of cerebrovascular dynamics and improve clinical management strategies for patients with lacunar strokes.

## Methods

The single center study at Shaare Zedek Medical Center was approved by the local Helsinki committee (IRB) without need for informed consent (0246-22-SZMC).

### Patient population

#### Inclusion criteria

**Fig. 1:**
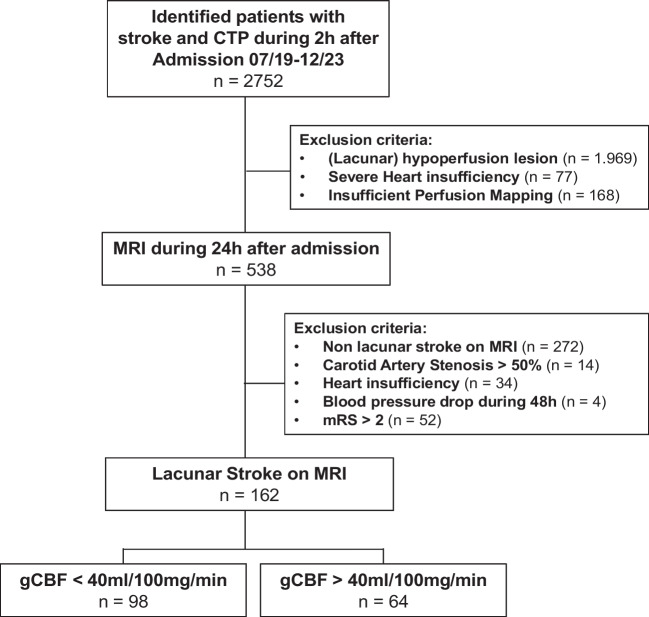
2752 patients were admitted between July 2019 until December 2023. All patients received CTP imaging during the first 2 h after admission to hospital. 538 of these patients performed an MRI scan during the first 24 h, and a total of 162 patients fulfilled all criteria

**Fig. 2:**
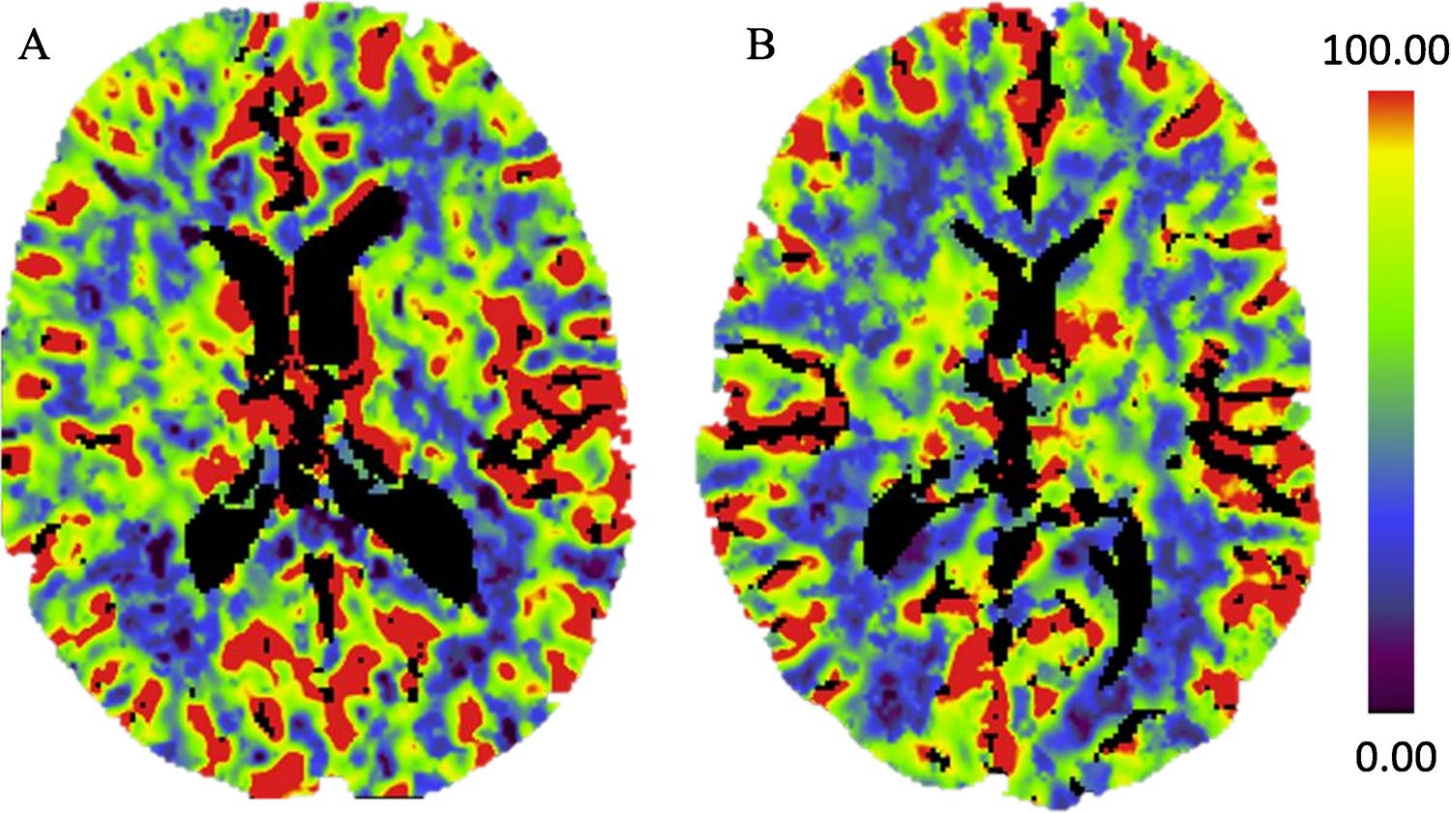
**A** and **B** show CBF mapping. **A** shows a patient with a normal gCBF of 45 ml/100 mg/min. **B** shows a patient with a gCBF of 28 ml/100 mg/min

Records for patients 18 years or older admitted between July 2019 and December 2023 for a first-ever ischemic stroke were screened for inclusion. Included patients were those of patients with CTP performed within the first 2 h of admission and within the first 8 h of stroke onset, and in addition diffusion weighted MRI of the brain performed within 24 h of admission. Records had to have met criteria for lacunar stroke by MRI imaging, defined as a restricted diffusion consistent with an acute ischemic lesion between 3 and 15 mm in size and located in either the basal ganglia, corona radiata, internal capsule, or pons (Figs. 1 and 2).

All patients received a standard of care with dual antiplatelet treatment, ruling out an effect of these medications to prevent further worsening or the main course of improving. Intracranial bleedings were not observed in this study.

#### Exclusion criteria

Patients who do not meet the imaging standards (movement, late arrival of bolus, more than one lacunar stroke on MRI), signs of moderate to severe leukoaraiosis (Fazekas III), uni- and bilateral carotid artery stenosis greater than 50% (NASCET), hypoperfusion lesion on CTP as known reason for worse outcome, signs of infection during the first 48 h, blood pressure drops greater than 60 mmHg or excessive above 220mmHg during the first 48 h that might contribute to worse outcome, treatment with tPA, known heart failure, pacemaker, prior large vessel stroke, hypercapnia greater than 50 mmHg, creatinine > 1.5 mg/dl, modified Rankin Score (mRS) greater than 2 before the index stroke, fever above 38.5 °C during the first 48 h, no signs of a single lacunar stroke on MRI.

### Radiologic assessment

**Fig. 3:**
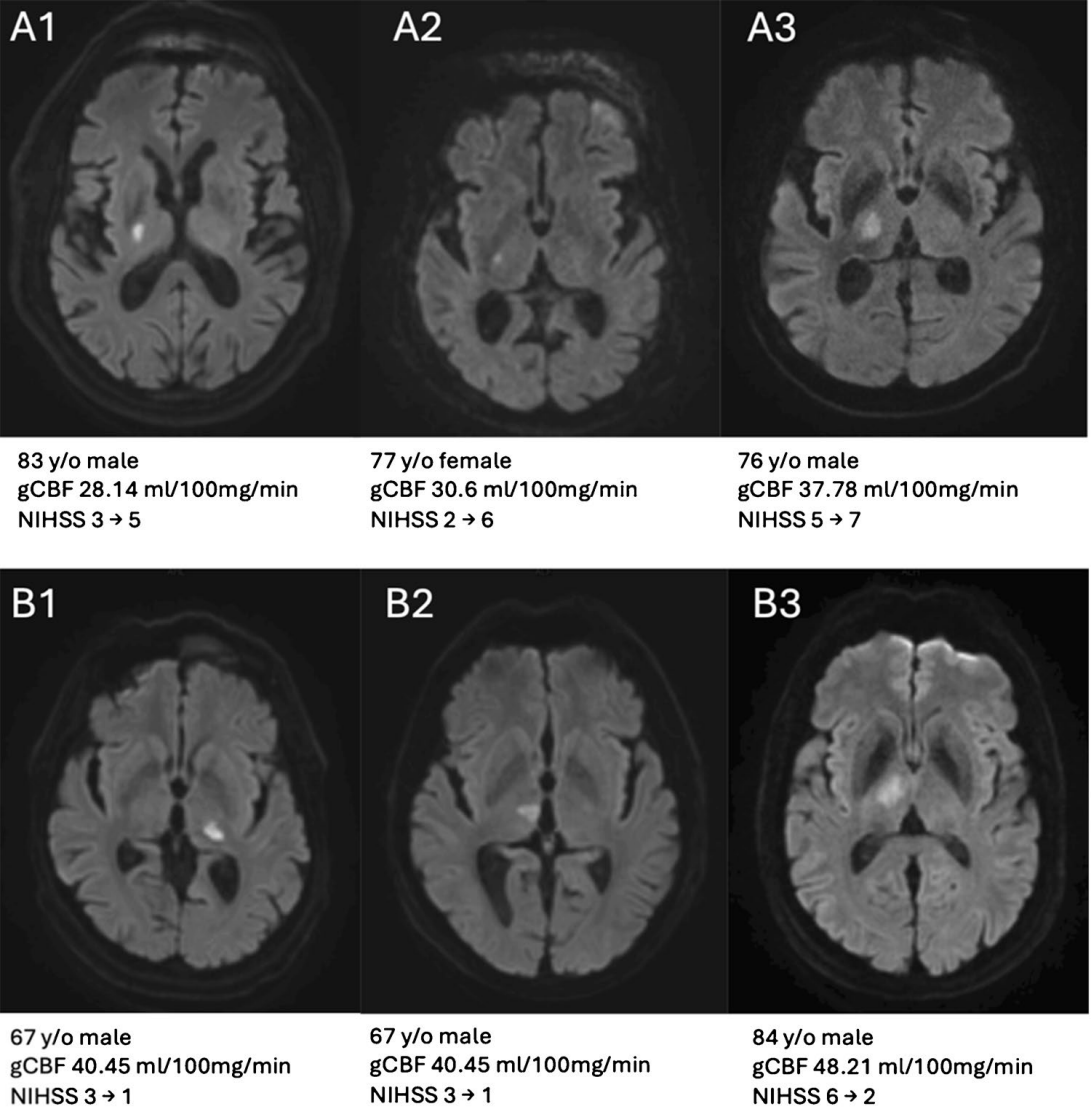
Various forms of basal ganglia lacunar infarctions are presented. Row A includes patients with low global cerebral blood flow (gCBF), exhibiting a higher rate of deterioration on the NIHSS scale. In Row B, similar infarctions are observed, but with normal gCBF levels above 40 ml/100 mg/min. All patients in Row A meet the criteria for early neurological deterioration, whereas patients in Row B show improvement over a 48-hour period. The latter group further improved to NIHSS 0 and were discharged home, while all patients in Row A underwent neurological rehabilitation

All patients performed a CTP (Fig. 3) during the first two hours from hospital admission and underwent an MRI study during the first 24 h. All patients had a venous access in the cubital vein with a flow of 80 ml/min. CTP was performed on a Siemens Somatom CT with 128 slices, contrast material was injected with a flow rate of 5.3 ml/sec and a total volume of 40 ml. The raw CTP data were analysed via GE AW ServerS and the functional imaging CT Brain Stroke application. The arterial input and venous output function were checked to rule out scans with motion or late contrast bolus arrival. Each scan consisted of 20 slices and each slice had a thickness of 5 mm. The automated mapping was transformed as range of interest and then cloned to CBF and CBV analysis. The process is fully automatic and offers a very precise inclusion of all brain tissue and exclusion of areas with cerebral spinal fluid or bone structure. Each of the 20 slices was analysed for CBF, and an average was collected as global Cerebral Blood Flow.

Subsequently, the global CBF of both cerebral hemispheres, cerebellum, and brain stem was measured and is further referred to as global Cerebral Blood Flow (gCBF).

The MRI scan was performed on a 3T Siemens Magnetom Vida during the first 24 h of stroke onset. The MRI was analysed by two Neuroradiologists as well as the attending Stroke Doctor. For evaluation of the stroke, the DWI sequence was analysed (Resolve DWI (b = 1000, TR:3300, TE:56.2).

Patients were further included with signs of Lacunar Stroke, and excluded with no signs of stroke, multiple strokes, **signs of leukoaraiosis**, or non-lacunar strokes.

### Clinical data collection

Patient data was retrieved from the local hospital data set, including demographic data, prior diseases and medications, blood pressure over the first 48 h, NIHSS on admission and every 12 h in the follow up. All patients received a dual antiplatelet therapy following the initial CT. Patients received 300 mg of acetylsalicylic acid as well as 300-600 mg of clopidogrel.

### Statistics

We performed analyses using SPSS. A one-sample Kolmogorov-Smirnov test was conducted to assess the patient population regarding gCBF. Further analyses included comparisons of means and univariate analysis for all parameters related to vascular risk factors, stroke location, and gCBF.

### Outcome assessment

Our primary outcome was presence of END, defined as an increase in the NIHSS ≥ 2 at 48 h after symptom onset.

## Results

A total of 2752 records admitted with ischemic lacunar stroke between 07/2019 and 12/2023 were screened. Of these, 538 were followed up after performing CTP (Fig. 2) and MRI (Fig. 3). A further 376 patients were excluded either due to exclusion criteria or not fulfilling inclusion criteria.

**Fig. 4:**
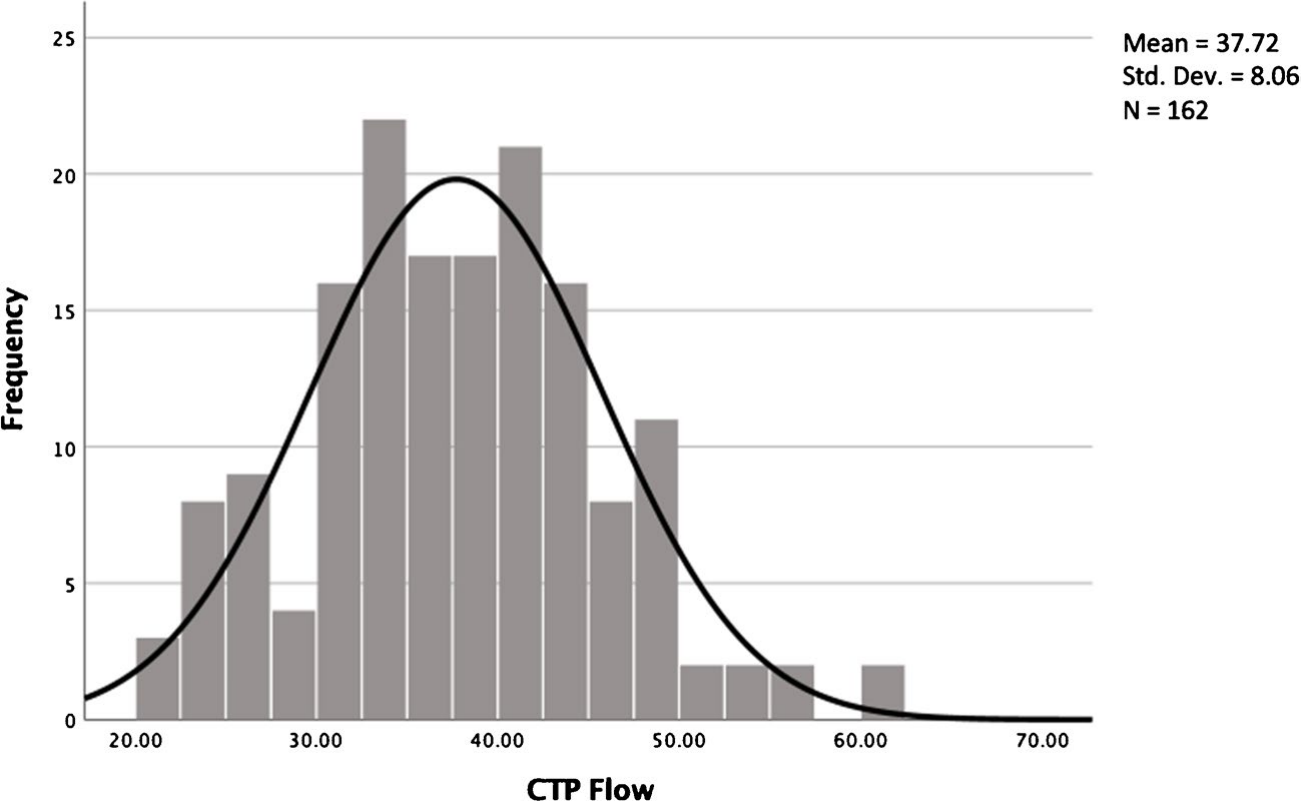
Distribution of gCBF of 162 patients. The mean gCBF is 37.72 with a SD+/- of 8.06. For further investigations, we chose 2 groups: normal gCBF above 40 ml/100 mg/min and a group with lower-than-normal gCBF (< 40 ml/100 mg/min)

To estimate the mean gCBF of our study population, we performed a one-sample Kolmogorov-Smirnov test. The test shows a mean gCBF of 37.72 ml/100 mg/min (Fig. 4) with a standard deviation of 8.06.

To evaluate our software (GE AW ServerS), we collected 30 patients that matched the age range in our study and received a CTP study due to wrong evaluation in the emergency department and no prove of acute restrictive lesion on MRI. The mean age was 69.1 and the average gCBF 47.3 ml/100 mg/min.

For the subsequent examinations, a cut off was chosen for normal gCBF with a flow above 40 ml/100 mg/min (64 patients) and a low CBF group with a flow below 40 ml/100 mg/min (98 patients).

Both groups show a similar age and gender differentiation. Vascular risk factors differ slightly in ischemic heart disease and diabetes with higher incidences in the low gCBF group. This group also shows a higher prevalence for strokes in the area of the corona radiata in comparison to more thalamic strokes in the normal gCBF group (Table 1).

**Table 1:** Patient characteristics for both groups with low and high gCBF

	gCBF < 40ml/100mg/min	gCBF > 40ml/100mg/min
	(*n* = 98)	(*n* = 64)
Characteristics		
Age	70.5	66.2
Male sex (%)	70.6	70.3
Vascular Riskfactors		
Art. Hypertension (%)	81.6	85.9
Ischemic Heart Disease (%)	28.6	15.6
Diabetes mellitus (%)	53.1	45.3
Smoking (%)	25.5	26.6
Dyslipidaemia (%)	60.2	64.1
Radiological Features (CTP)		
Global CBF (ml/100mg/min)	32.1	45.5
Stroke localisation on MRI		
Basal Ganglia (%)	16.3	25.0
Capsula Interna (%)	16.3	7.8
Basal Ganglia w/o Thalamus (%)	11.2	4.7
Corona Radiata (%)	35.7	35.9
Pons (%)	20.4	26.6
Outcome after 48 hours		
Worsening ≥ 2 points on NIHSS (no / %)	37 (37.8)	2 (3.1)
Worsening 1 point on NIHSS (no / %)	26 (26.5)	7 (10.9)
No changes in NIHSS (no / %)	29 (29.6)	14 (21.9)
Improving (no / %)	6 (6.1)	41 (46.5)

Both groups show a similar amount of age and gender differentiation. Vascular Risk factors are similar with a slightly higher rate of dyslipidaemia and diabetes in the low gCBF group. The low gCBF group shows more infarctions in the area of corona radiata, the high gCBF group shows a higher prevalence of thalamic stroke

Both patient groups were analysed for NIHSS scores on admission and followed up over the course of 48 h with NIHSS scores every 12 h. The average initial NIHSS was 4.2 (SD+/- 3.39) for the normal gCBF group and 4.2 (SD+/- 3.07) for the low gCBF group. Patients with a normal gCBF showed an improvement to NIHSS 2.9 (SD+/- 3.44 (*p* < 0.001)), while the other group shows slight worsening to 5.3 (SD+/- 3.68 (*p* < 0.001)) (Table 2).

**Table 2:** We conducted univariate analysis of all vascular risk factors, stroke localization, and global cerebral blood flow in relation to outcome

Univariate analysis *p* < 0.05		
	gCBF < 40ml/100mg/min	gCBF > 40ml/100mg/min
	(*n* = 98)	(*n* = 64)
Characteristics		
Age	0.372	0.61
Gender	0.119	0.419
Art. Hypertension	0.573	0.654
Diabetes mellitus	0.443	0.139
Smoking	0.469	0.813
Dyslipidaemia	0.741	0.366
Stroke Localisation	0.372	0.717
Lactate	0.147	0.284
Initial Blood Pressure	0.624	0.958
Creatinine	0.91	0.948
Blood Glucose	0.711	0.836
Global CBF (ml/100mg/min)	<0.001	<0.001

None of the vascular risk factors were found to be prognostic factors for outcome. Neither lactate, glucose level, nor blood pressure were associated with outcome. However, global CBF exhibited a high level of significance as a predictor of worsening of stroke symptoms within the next 48 h

To differentiate between the two groups and different sites of the lacunar strokes, we also analysed the outcome for each individual stroke site.

Specifically in the low gCBF group we observed an average a worsening of at least 1 point in all areas with highest rates of worsening in corona radiata and LPI. The area of thalamus shows non-significant changes in the NIHSS score.

Extrapolating only patients with END (worsening of at least 2 points on the NIHSS scale), 2 (3.1%) patients in the normal gCBF group fulfil the criteria versus 37 (37.8%) patients in the low gCBF group.

Out of the 2 patients showing END in the normal gCBF group, one presented with a stroke in the area of thalamus, the other in the area of internal capsule, both with high initial NIHSS. These patients showed a worsening of 2 and of 3 points on the NIHSS scale.

Regarding improvement, only 6 patients (6.1%) with low gCBF improved by at least 2 points on the NIHSS scale. In comparison 46.8% of normal gCBF improved by at least 2 points and additional 17.4% improved by 1 point.

Univariate analysis proved no significance for all parameters on admittance of the patient. After 48 h there is still no significance for initial blood pressure (*p* = 0.958), creatinine (*p* = 0.948), blood glucose (*p* = 0.836), lactate (*p* = 0.284) and stroke site (*p* = 0,185). Only gCBF proves to be an independent marker for outcome in lacunar strokes (*p* < 0.001).

## Discussion

In this retrospective single-center study, we could successfully demonstrate that gCBF serves as a prognostic marker for outcome following lacunar infarction. The study’s reliability is enhanced by our strict reliance on MRI for patient selection, ensuring the inclusion of only those clearly defined as lacunar strokes ranging from 2 to 15 mm in size and location in the typical areas. Subcortical lesions outside the corona radiata were not included in this study.

In our cohort, all patients received the same treatment to reduce confounders. All patients performed the CTP on the same CT scanner, with the same setting and the same vascular access (18G access in basilic or cephalic vein). All patients with blood pressure instabilities were excluded as well as patients with signs of infection as known course for worsening of neurological symptoms.

All patients received a standard of care with dual antiplatelet treatment, ruling out an effect of these medications to prevent further worsening or the main course of improving. Intracranial bleedings were not observed in this study.

Regarding the patient population, we have seen a higher incidence of diabetes mellitus and ischemic heart disease in the lower gCBF group. The high rate of male patients in both groups is in accordance with other studies [18, 19] showing a higher prevalence for lacunar strokes in the male population.

Vynckier et al. [7] showed the highest incidence of lacunar strokes in specific brain regions such as the internal capsule, striatocapsular, and pons, followed by the thalamus, with a relatively equal distribution in other areas. Our study validated and expanded upon these findings by identifying specific patient characteristics that were more likely to experience deterioration. In both groups, normal gCBF and low gCBF, we observed a worse outcome in the regions of capsula interna and pons, though to varying degrees.

**Fig. 5:**
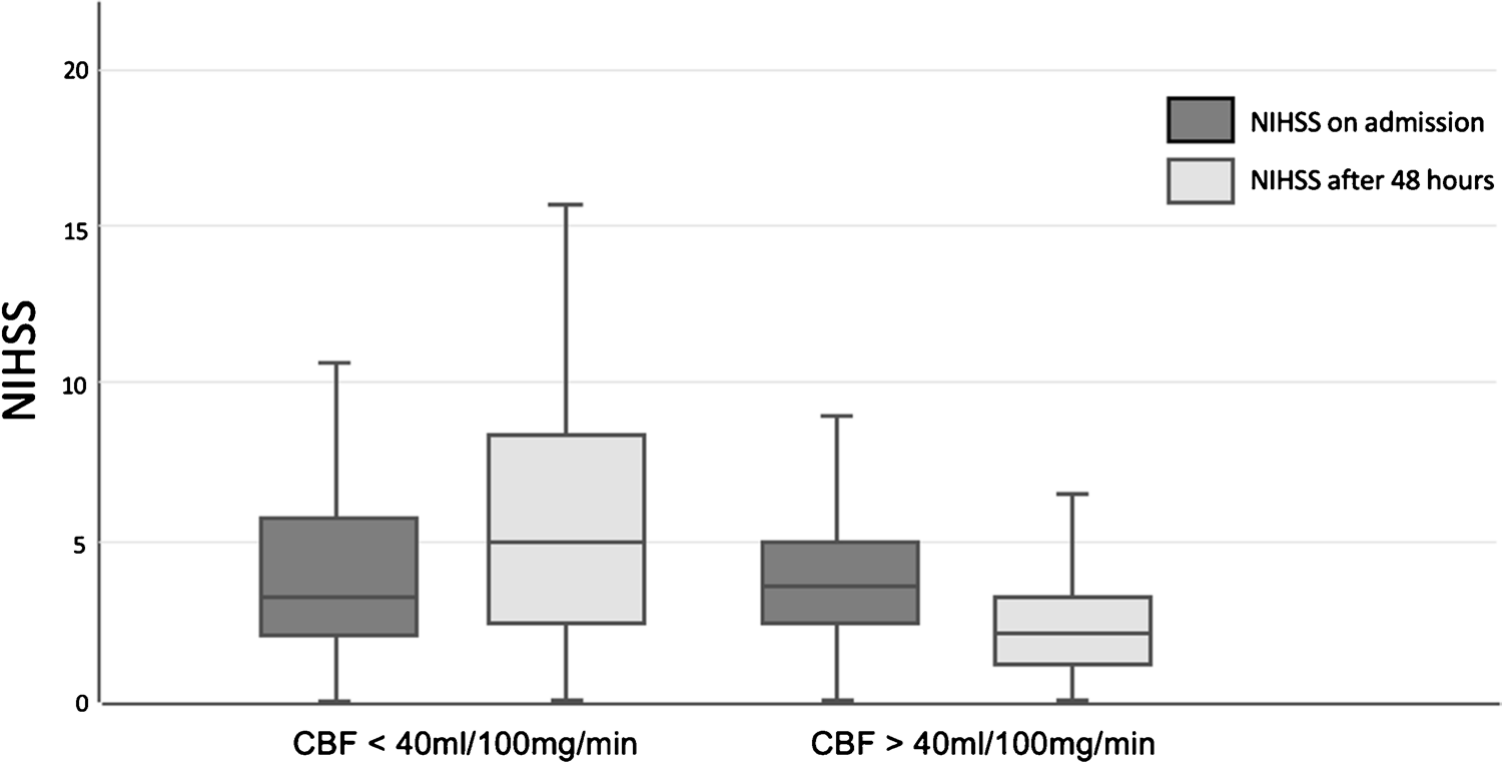
Comparison of both patient groups. The normal gCBF group shows an improvement from NIHSS 4.2 (SD+/- 3.39) points by 1.3 points to NIHSS 2.9 (SD+/- 3.44). The low gCBF group shows an initial similar average NIHSS with 4.2 (SD+/- 3.07) points. Over the course of 48 h we see a worsening by 1.3 points to 5.3 (SD+/- 3.68) Both results reach a high significant with *p* < 0.001

Patients with a normal gCBF only met criteria for END in 3.1%, 64.3% showed improvement and 21.9% stayed stable on a low NIHSS between 0 and 2. The mean improvement for this group was 1.4 points over the course of 48 h (Fig. 5).

**Fig. 6:**
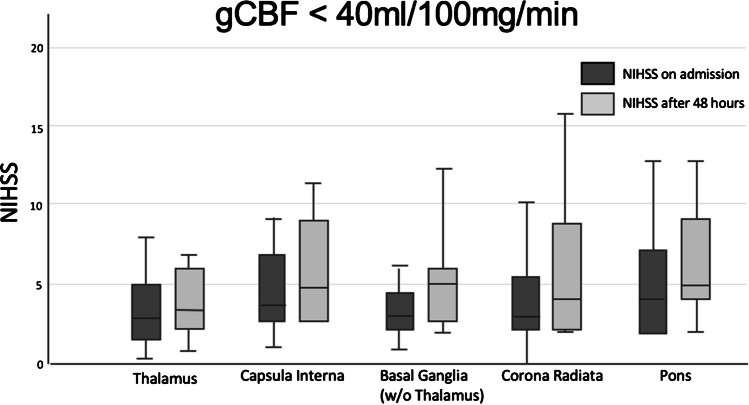
The boxplot diagram shows the mean outcome of patients with a gCBF below 40 ml/100 mg/min. These patients show on average a worsening after 48 h. The changes in NIHSS: thalamus 3.6 -> 3.4, capsula interna 4.4 -> 5.3, basal ganglia 4.0 -> 5.1, corona radiata 4.2 -> 5.6, LPI 4.7 -> 6.5. All results are significant in the worsening but the stroke location itself shows no significance

**Fig. 7:**
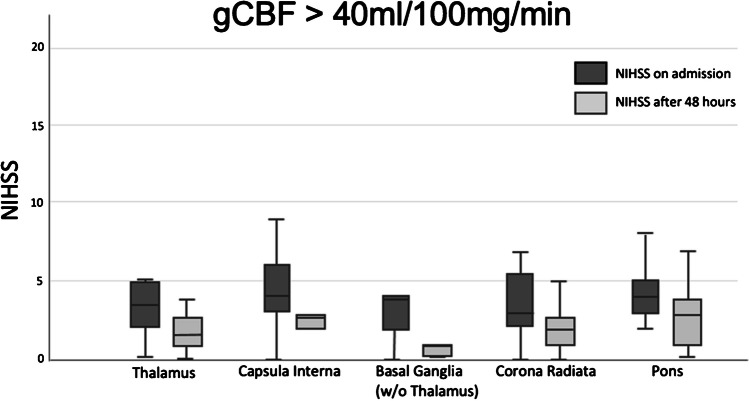
The boxplot diagram shows the mean outcome of patients with a gCBF above 35 ml/100 mg/min. These patients show on average a slight improvement in NIHSS Score after 48 h. The changes in NIHSS: thalamus 4.1 -> 3.1, capsula interna 4.4 -> 2.2, basal ganglia 2.7 -> 0.7, corona radiata 4.17 -> 2.8, LPI 4.7 -> 3.3. All results are significant in the worsening but the stroke location itself shows no significance

Conversely, the low gCBF group showed an average worsening of 1 point on the NIHSS scale, with higher rates of deterioration observed specifically in the capsula interna and pons. A total of 37.8% could be classified with END and only 6.1% showed improvement. In total, 64.3% showed neurological deterioration. It is worth mentioning that a lower gCBF correlates with a higher rate of worsening and END (Fig. 6). A cut off at 40 ml/100 mg/min shows a mean worsening of 1.1. A cut off at 35 ml/100 mg/min shows a mean worsening of 1.7 points on the NIHSS scale with a rate of END at 44.4%. With further reduction of gCBF the rate of END rises (Fig. 7).

Part of the worsening for all patients in the areas of capsula interna and pons can be attributed to the findings of Nagakane et al. [19], who demonstrated that regional edema and an increase in stroke size have an impact on the corticospinal tract, leading to a significant deterioration. Reduced cerebral blood flow may contribute to the exacerbation of edema. This is further corroborated by Yamada et al. [20], who demonstrated that regional changes in CTP within the lacunar stroke area serve as predictors of poor outcomes. However, our study managed to establish predictability even in the absence of signs of lacunar stroke on CTP.

The idea of cardiovascular influence on outcome for ischemic stroke has been discussed at length [21]. Tsivgoulis et al. [22] postulated 2008 the idea of a cerebrovascular reserve (CRV). They describe 3 stages with normal flow in Stage 0 and oligemia and ischemic stroke in Stage 2 that determines the failure of cerebral autoregulation. Stage 1 describes the maximum of cerebral autoregulation with increasing Mean Transit Time, CBF and CBV.

CBF is an established parameter in CTP used to estimate the size of stroke. In healthy individuals, the average CBF ranges from 40 to 50 ml/100 mg/min [13]. However, the patients in our study demonstrated a mean gCBF of 32.1 ml/100 mg/min in the low gCBF group in contrast to 45.5 ml/100 mg/min in the normal gCBF group.

Viewing CBF as part of cerebral autoregulation can offer insights into the natural course of lacunar strokes. With normal gCBF, improvement over time is observed. However, in cases where low gCBF is associated with diminished CRV, patients tend to experience worse outcomes regarding NIHSS. Earlier MRI studies could prove the concept of a penumbral area also around lacunar strokes [23, 24] with worsening over time. We estimate that specifically individuals with atheromatous stenosis of perforating arteries or endothelial dysfunction might suffer more due to reduced blood flow in the stroke-affected region, leading to increased edema and stroke size.

In the context of adaptive autoregulation, cerebral autoregulation can elevate blood flow to ensure sufficient circulation, thereby reducing edema and maintaining a stable penumbra, which prevents further deterioration. Conversely, low gCBF can lead to a worsening as it contributes to the development of edema and further progression of the stroke.

As a single-center study, there are limitations regarding results. Imaging was performed on one single CT and MRI and data acquisition of CTP was archived on commercially available software.

With this study we confirmed previously known data about the general worse outcomes associated with lacunar strokes in the areas of internal capsule and pons. Additionally, we proved that lower than normal gCBF is an independent factor to prognose worse outcome in all lacunar infarctions sites.

## Conclusion

Global Cerebral Blood Flow serves as an independent marker for a worse outcome in cases of lacunar stroke. This offers a novel screening tool for the early assessment of patients at high risk of experiencing a deterioration in their stroke symptoms within the first 48 h following the onset of the stroke.

## Data Availability

The datasets generated and analyzed during the current study are available from the corresponding author upon reasonable request.
